# Duration and mode of delivery: Does maternal sleep matter?

**DOI:** 10.1002/ijgo.70673

**Published:** 2025-11-20

**Authors:** Hilla Parttimaa, E. Juulia Paavonen, Laura Perasto, Hasse Karlsson, Linnea Karlsson, Päivi Polo‐Kantola

**Affiliations:** ^1^ Department of Obstetrics and Gynecology Turku University Hospital and University of Turku Turku Finland; ^2^ Department of Public Health Finnish Institute for Health and Welfare Helsinki Finland; ^3^ Pediatric Research Center, Child Psychiatry University of Helsinki and Helsinki University Hospital Helsinki Finland; ^4^ FinnBrain Birth Cohort Study, Turku Brain and Mind Center, Department of Clinical Medicine University of Turku and Turku University Hospital Turku Finland; ^5^ Center for Population Health Research Turku University Hospital and University of Turku Turku Finland; ^6^ Department of Public Health University of Turku and Turku University Hospital Turku Finland; ^7^ Department of Child Psychiatry Turku University Hospital and University of Turku Turku Finland

**Keywords:** delivery, delivery type, duration of delivery, insomnia, labor, pregnancy, sleep

## Abstract

**Objective:**

Poor sleep quality in late pregnancy has been identified as a risk factor for operative deliveries. In this study, we investigated the associations between maternal sleep throughout pregnancy and its impact on both the duration and mode of delivery.

**Methods:**

We enrolled 3141 mothers and assessed sleep at four time points during pregnancy (early, mid‐, and late pregnancy, and at delivery) using the Basic Nordic Sleep Questionnaire. Delivery data were obtained from the Finnish Birth Registry. Both longitudinal and cross‐sectional analyses were performed, adjusting for confounding factors.

**Results:**

In mid‐pregnancy, poor sleep quality was associated with a longer first stage (436 vs. 399 min, *P* = 0.027) and overall duration of delivery (468 vs. 431 min, *P* = 0.034). In late pregnancy, short sleep duration (<6 h) (adjusted odds ratio [aOR] 2.57, 95% confidence interval [CI]: 1.15–5.72, *P* = 0.021) and poor sleep quality (aOR 1.68, 95% CI: 1.17–2.41, *P* = 0.005) were linked to an increased likelihood of unplanned cesarean section. Poor sleep quality (aOR 2.60, 95% CI: 1.52–4.46, *P* < 0.001) and frequent nocturnal awakenings (aOR 1.72, 95% CI: 1.01–2.89, *P* = 0.042) were associated with planned cesarean section, while habitual awakenings also increased the risk of vacuum extraction (aOR 1.45, 95% CI: 1.07–1.96, *P* = 0.018). At delivery, poor sleep quality remained associated with planned cesarean section (aOR 1.99, 95% CI: 1.04–3.82, *P* = 0.037). In longitudinal analyses, persistent insomnia and sleepiness were not associated with duration or mode of delivery.

**Conclusion:**

Maternal sleep is associated with the mode, but not with the duration of the delivery. Poor and short sleep during late pregnancy is related to instrumental deliveries.

**Abbreviations**
aOR: adjusted odds ratio
Aβ: adjusted correlation coefficient
BMI: body mass index
BNSQ: Basic Nordic Sleep Questionnaire
CI: confidence interval
EMM: estimated marginal means
gwk: gestational week/weeks
SD: standard deviation

## 1 INTRODUCTION

The majority of pregnant mothers experience deterioration of sleep quality.^1^ Insomnia and its variants are the most common symptoms, accompanied by daytime sleepiness and fatigue.^2^ Compared to the pre‐pregnancy, sleep is already altered in the first trimester, with increased total sleep time, fatigue, and napping.^3^, ^4^, ^5^ From the second trimester onwards, total sleep time typically decreases and nocturnal awakenings become more frequent.^6^, ^7^ Sleep disturbances peak in late pregnancy, and nearly 80% of women report markedly poorer sleep quality.^1^, ^4^, ^8^ These changes result from multiple factors, including hormonal shifts, physio‐anatomical adaptations, and psychological factors.^1^, ^2^, ^3^, ^9^, ^10^ Additionally, nocturnal breathing disturbances, particularly snoring, are common and contribute to fragmented sleep.^11^, ^12^, ^13^

Maternal sleep disturbances may influence delivery outcomes. Adequate sleep duration and good sleep quality have been associated with more favorable delivery courses.^14^ For example, Zafarghandi et al. reported that sleeping more than 8 h per night in late pregnancy was linked to spontaneous vaginal delivery,^14^ while Lee et al. found that sleeping less than 6 h per night in the week preceding delivery increased both labor duration and cesarean section risk.^15^ Shorter sleep has also been associated with prolonged induction‐to‐delivery intervals.^16^ Furthermore, some studies suggest that maternal poor sleep quality,^17^, ^18^, ^19^ short sleep,^16^ snoring,^19^ and fatigue^20^ are related to higher cesarean section rates. However, other studies have not confirmed these associations, especially when adjusting for confounders such as mental health and socioeconomic factors.^12^, ^21^

The aim of the present study was to investigate the associations between maternal sleep during pregnancy and both delivery duration and mode, using comprehensive adjustments for potential confounders. We hypothesized that sleep disturbances would be linked to longer vaginal deliveries and an increased likelihood of operative delivery, particularly cesarean section.

## 2 MATERIALS AND METHODS

### 2.1 Study design and settings

Our study was a part of a large prospective Finnish longitudinal birth cohort. The study design is described in detail elsewhere.^22^ Participants were recruited at early pregnancy ultrasound visits. Eligibility required adequate Finnish language skills, informed consent, and willingness to participate.

### 2.2 Participants and data enrollment

**FIGURE 1:**
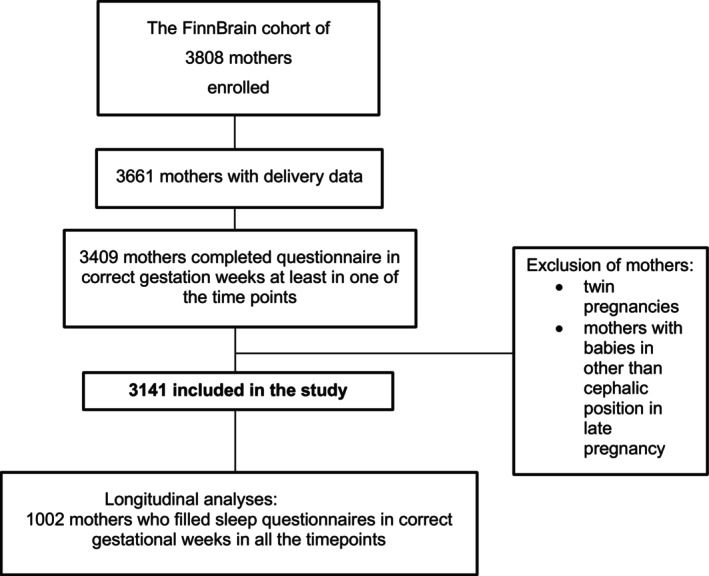
Flow chart of the study.

Questionnaires were administered at gestational weeks (gwk) 14, 24, and 34, and within 0–7 days postpartum. Responses were received at mean gwks 15, 25, and 35, and 2 days after delivery. Of 3808 enrolled mothers, 3499 completed at least one questionnaire. Delivery data were collected from hospital records and national registries. Exclusion criteria were multiple pregnancies, non‐cephalic presentation, or missing delivery or sleep data, resulting in a final sample of 3141 participants (see Figure 1).

The basic characteristics of the study cohort are shown in Table 1. Sociodemographic factors included age (years), parity (nulliparous/multiparous), and education (low/intermediate/high). The health behavior factors considered were body mass index (BMI, calculated as weight in kilograms divided by the square of height in meters) at the beginning of the pregnancy and smoking (yes/no). Depressive symptoms were evaluated with the Edinburgh Postnatal Depression Scale (EPDS)^23^ and anxiety with a Symptom Checklist‐90/Anxiety Scale (SCL‐90/anxiety scale)^24^ at the early pregnancy time‐point and were used as covariates. Gestational weeks at birth were collected from hospital records.

**TABLE 1:** Basic characteristics.

	*n* (%)	Mean	SD	Range
Mother's age	3141	30.17	4.69	17–46
BMI	3137	24.64	4.92	15.62–60.61
EPDS (early pregnancy)	2504	5.18	4.01	0–27
SCL (early pregnancy)	2405	3.35	3.97	0–33
*Education*	2549			
Low	952 (37.3)			
Intermediate	748 (29.3)			
High	849 (33.3)			
*Smoking*	3133			
Yes	412 (13.2)			
No	2721 (86.8)			
*Parity*	2544			
Nullipara	1278 (50.2)			
Multipara	1266 (49.8)			
*Delivery week*				
Continuous	3141	39.86	1.56	24.71–42.43
<32 h	10			
<37 h	116			
*Delivery duration (min)*	2702			
First phase	2712	441.77	283.08	8–2790
Second phase	2743	30.46	27.91	1–240
Total duration	2702	472.12	294.23	14–2892
*Delivery type*	3141			
Spontaneous	2430 (77.4)			
Vacuum	324 (10.3)			
Unplanned cesarean	274 (8.7)			
Planned cesarean	113 (3.6)			

*Note*: BMI, calculated as weight in kilograms divided by the square of height in meters.

Abbreviations: BMI, body mass index; EPDS, Edinburgh postnatal depression scale; SCL, symptom checklist (anxiety); SD, standard deviation.

### 2.3 Sleep assessment and sleep data

Maternal sleep during the past month was assessed with 11 questions from the Basic Nordic Sleep Questionnaire (BNSQ), covering sleep duration, sleep need, general sleep quality, insomnia symptoms (difficulties falling asleep, nocturnal and early morning awakenings), snoring, morning and daytime sleepiness, and napping. General sleep quality was rated on a five‐point scale. Sleep duration (<6 vs. ≥6 h) and sleep loss (<2 vs. ≥2 h) were categorized. General sleep quality, awakenings, and snoring were dichotomized. The insomnia score (range: 5–25) was derived by summing five items; scores ≥16 indicated insomnia symptoms. The sleepiness score (range: 3–15) summed three items; scores ≥12 indicated sleepiness symptoms (Table 2).

**TABLE 2:** Sleep assesment in the study.

Variable	Assessment	Scoring/categorization
Sleep duration	Hours per night	Categorized: <6 h vs. ≥6 h
Sleep need	Hours per night	–
Sleep loss	Sleep need minus sleep duration	Categorized: <2 h vs. ≥2 h
General sleep quality	5‐point scale: 1 = good to 5 = poor	Dichotomized: 1–3 = good, 4–5 = poor
Difficulties falling asleep	5‐point frequency scale (never to daily)	–
Nocturnal awakenings per week	5‐point frequency scale (never to daily)	–
Nocturnal awakenings per night	5‐point scale: 1 = none to 5 = ≥5 awakenings	Dichotomized: 1–3 = occasional, 4–5 = habitual
Too early morning awakenings	5‐point frequency scale (never to daily)	–
Snoring	5‐point frequency scale (never to daily)	Dichotomized: 1–3 = occasional, 4–5 = habitual
Morning sleepiness	5‐point frequency scale (never to daily)	–
Daytime sleepiness	5‐point frequency scale (never to daily)	–
Napping	5‐point frequency scale (never to daily)	–
Insomnia score	Sum of 5 variables (general sleep quality, difficulties falling asleep, nocturnal awakenings per week and per night, too early awakenings)	Range: 5–25; ≥16 = insomnia symptoms
Sleepiness score	Sum of 3 variables (morning sleepiness, daytime sleepiness, napping)	Range: 3–15; ≥12 = sleepiness symptoms

The primary outcome variables were the duration of delivery among mothers with spontaneous vaginal deliveries (phase I, phase II, and total duration in minutes) and the mode of delivery, categorized as spontaneous vaginal delivery, vacuum extraction, unplanned cesarean section, or planned cesarean section.

### 2.4 Statistical analysis

#### 2.4.1 Cross‐sectional analyses at four time points

First, descriptive analysis was performed, and the results are expressed by means and standard deviations (SD) or the number of cases and percentages. The distribution of duration of delivery‐data was skewed, so a logarithmic and square root transformations were used to approximately conform data to normality. First phase duration and total delivery duration were transformed using square root transformation and second phase duration was transformed using log_10_‐transformation. Few outliers were also excluded from the analysis. Of note is, that only mothers delivering vaginally were included in the analysis concerning delivery duration.

Then, associations between sleep variables and the duration of delivery were analyzed by general linear models, and associations between sleep variables and the mode of delivery were evaluated by logistic regression analysis separately at each pregnancy point. All models were adjusted for age, parity, education, BMI, smoking, and depressive and anxiety symptoms, as well as gwk at delivery. Estimates from adjusted linear models are expressed as Aβ as well as the estimated marginal means (EMM) with back transforming the estimates to the original scale (min). Estimates from adjusted logistic regressions are expressed as adjusted odds ratios (aORs). The 95% confidence intervals (CI) were calculated for estimates.

#### 2.4.2 Longitudinal setting for the study of persistent insomnia and sleepiness symptoms

In the longitudinal setting, only mothers who had filled questionnaires at all four time points were taken into the analysis. Longitudinal setting was conducted to insomnia score (*n* = 1000) and sleepiness score (*n* = 1002). The cutoff points were 16 for the insomnia score and because of few cases in sleepiness score ≥ 12, the cutoff point ≥10 was chosen in longitudinal setting. Of note is, that only mothers delivering vaginally were included in the analyses concerning delivery duration.

Mothers were categorized into three groups to represent insomnia or sleepiness symptoms lasting throughout the whole pregnancy: (1) Persistent low scores: the scores were lower than the cutoff point in all four time points (insomnia score, *n* = 312; sleepiness score, *n* = 442). (2) Persistent high scores: the scores were higher or the same as the cutoff point after mid pregnancy (insomnia score, *n* = 147; sleepiness score, *n* = 137). (3) Intermediate scores: the scores were not low or high throughout the pregnancy but varied between the time points (insomnia score, *n* = 541; sleepiness score, *n* = 423).

Associations between the formed score variables and the duration of delivery were analyzed with a general linear model, and the mode of delivery with binary logistic regression model. All models were adjusted for age, parity, education, BMI, smoking, and depressive and anxiety symptoms, as well as gwk at delivery. Mothers with persistent high scores and intermediate scores throughout the pregnancy were compared with mothers with persistent low scores.

In the analyses regarding delivery types, the number of mothers in each group was too small when divided into four delivery types (spontaneous/vacuum extraction/unplanned and planned cesarean sections). Therefore, in the longitudinal setting, the delivery types were dichotomized to vaginal (spontaneous and vacuum extraction) and cesarean sections (unplanned and planned cesarean sections). Statistical analyses were made with SPSS Statistics version 27 and with R4.1.0 (R Core Team, 2021). Figures were made with R using the library ggplot2.

## 3 RESULTS

Delivery characteristics are presented in Table 1. In this cohort, 50.2% of mothers were nulliparous. The mean gestational age at delivery was 39.9 weeks (SD 1.6; range: 24.7–42.4). The mean durations of labor were: first stage, 7 h 22 min; second stage, 30 min; and total duration, 7 h 52 min. Overall, 87.7% (*n* = 2754) had a vaginal delivery (spontaneous or vacuum‐assisted), while 12.3% (*n* = 387) underwent cesarean section (planned or unplanned).

Maternal sleep characteristics are summarized in Table 3. The insomnia score increased significantly over the course of pregnancy (early pregnancy vs. delivery, *P* < 0.001). Although average sleep duration declined only marginally (*P* < 0.001), the proportion of short sleepers rose toward the end of pregnancy (*P* < 0.001). Reports of poor general sleep quality and frequent nocturnal awakenings also increased significantly (*P* < 0.001). The prevalence of habitual snoring showed a slight but significant rise (*P* < 0.001).

**TABLE 3:** Maternal sleep.

	*n* (%)	Mean	SD	Range
*Insomnia score (continuous)*				
Early pregnancy	2473	12.68	3.43	5–24
Mid pregnancy	2263	12.97	3.38	5–24
Late pregnancy	2128	15.05	3.37	5–25
Delivery	1953	16.40	3.47	5–25
*Sleep duration (continuous)*				
Early pregnancy	2487	7.92	1.01	4–14
Mid pregnancy	2274	7.84	0.99	3–13
Late pregnancy	2132	7.88	1.15	3–15
Delivery	1849	7.74	1.31	2–13
*Sleepiness score (continuous)*				
Early pregnancy	2479	7.92	2.52	3–15
Mid pregnancy	2263	8.32	2.75	3–15
Late pregnancy	2122	8.43	2.64	3–15
Delivery	1948	8.65	2.71	3‐–5

	Early *n* (%)	Mid *n* (%)	Late *n* (%)	Delivery *n* (%)
*Sleep quality*	2378	1997	2100	1302
Poor	353 (14.8)	317 (15.9)	627 (29.9)	527 (40.5)
Good	2025 (85.2)	1680 (84.1)	1473 (70.1)	775 (59.5)
*Sleep loss*	2436	2229	2082	1801
≥2 h	562 (23.1)	515 (23.1)	447 (21.5)	464 (25.8)
<2 h	1874 (76.9)	1714 (76.9)	1635 (78.5)	1337 (74.2)
*Awakenings per night*	2372	1994	2098	1302
≥3	316 (13.3)	299 (15.0)	713 (34.0)	527 (40.5)
<3	2056 (86.7)	1695 (85.0)	1385 (66.0)	775 (59.5)
*Snoring*	2339	1976	2060	1288
Habitual	171 (7.3)	170 (8.6)	284 (13.8)	208 (16.1)
Occasional	2168 (92.7)	1806 (91.4)	1776 (86.2)	1080 (83.9)
*Sleep duration*	2487	2274	2132	1849
<6 h	37 (1.2)	43 (1.9)	57 (2.7)	110 (5.9)
≥6 h	2450 (98.5)	2231 (98.1)	2075 (97.3)	1739 (94.1)
*Insomnia score*	2473	2263	2128	1953
≥16	480 (19.4)	481 (21.3)	971 (43.1)	1159 (59.3)
<16	1993 (80.6)	1782 (78.7)	1211 (56.9)	795 (40.7)
*Sleepiness score*	2479	2263	2122	1948
≥12	313 (12.6)	190 (8.4)	286 (13.5)	313 (16.1)
<12	2166 (87.4)	2073 (91.6)	1836 (86.5)	1635 (83.9)

**FIGURE 2:**
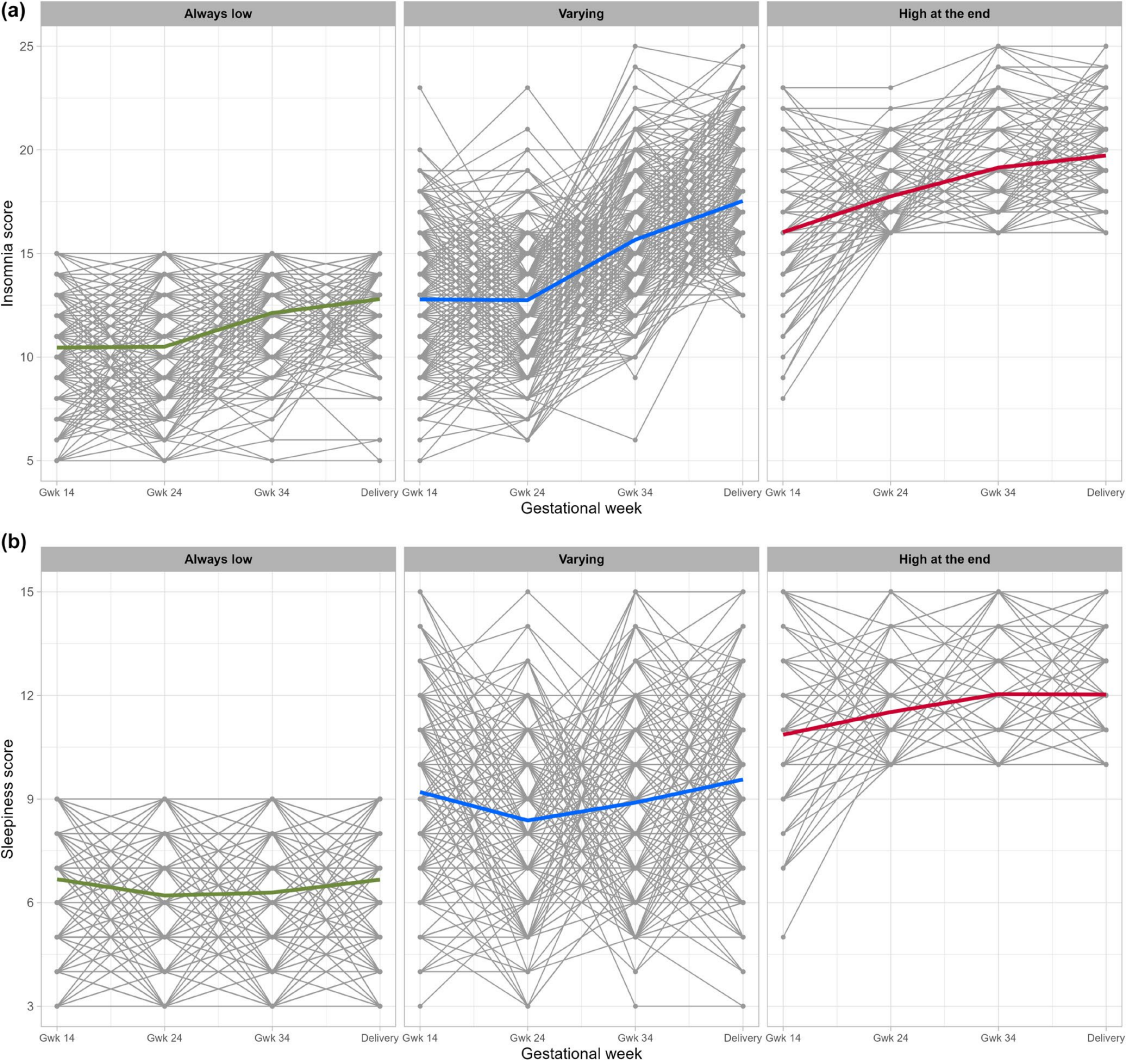
(a) Insomnia scores across pregnancy in mothers answering questionnaires in all the timepoints. Mothers were categorized into three groups to represent insomnia symptoms lasting throughout the whole pregnancy: (1) persistent low scores, (2) persistent high scores, (3) intermediate scores, *n* = 1000. (b) Sleepiness scores across pregnancy in mothers answering questionnaires in all the timepoints. Mothers were categorized into three groups to represent sleepiness symptoms lasting throughout the whole pregnancy: (1) persistent low scores, (2) persistent high scores and (3) intermediate scores, *n* = 1002.

Persistent insomnia and sleepiness scores throughout pregnancy are illustrated in Figure 2a,b. All significant associations between maternal sleep and both delivery duration and mode are presented in Table 4, with full results available in the Supporting Information Appendix Tables 1a–d and 2a–d.

**TABLE 4:** Main findings of the study.

Time‐point	Sleep parameter	Outcome
Mid‐pregnancy	Poor sleep quality	Longer first stage of labor (436 vs. 399 min, *P* = 0.027)
		Longer total duration (468 vs. 431 min, *P* = 0.034)
Late pregnancy	Short sleep (<6 h)	Increased risk of unplanned cesarean (aOR 2.57, *P* = 0.021)
	Poor sleep quality	Increased risk of unplanned cesarean (aOR 1.68, *P* = 0.005)
	Poor sleep quality	Increased risk of planned cesarean (aOR 2.60, *P* < 0.001)
	Frequent nocturnal awakenings	Increased risk of planned cesarean (aOR 1.72, *P* = 0.042)
	Frequent nocturnal awakenings	Increased risk of vacuum extraction (aOR 1.45, *P* = 0.018)
At delivery	Poor sleep quality	Increased risk of planned cesarean (aOR 1.99, *P* = 0.037)
Longitudinal	Persistent insomnia/sleepiness	No significant associations with duration or delivery mode

### 3.1 Duration of delivery

The adjusted cross‐sectional associations between sleep variables and the duration of delivery are presented in detail in the Supporting Information Appendix. Women reporting poor sleep quality during mid‐pregnancy had a longer first stage (adjusted mean minutes [EMM] 436 vs. 399; aOR 0.90, 95% CI: 0.10–1.70, *P* = 0.027) and longer total duration of delivery (EMM 468 vs. 431; aOR 0.85, 95% CI: 0.07–1.64, *P* = 0.034). No other significant associations were observed.

In longitudinal analyses, persistent high or intermediate insomnia scores throughout pregnancy were not associated with the duration of delivery after adjustment. Specifically, no associations were found for: (1) First stage: persistent high versus low insomnia scores (*P* = 0.626), intermediate versus low (*P* = 0.552). (2) Second stage: persistent high versus low (*P* = 0.508), intermediate versus low (*P* = 0.473). (3) Total duration: persistent high versus low (*P* = 0.802), intermediate versus low (*P* = 0.669). Regarding sleepiness, no significant associations emerged between persistent high or intermediate sleepiness scores and the duration of delivery in any stage after adjustment: (1) First stage: high versus low (*P* = 0.396), intermediate versus low (*P* = 0.748). (2) Second stage: high versus low (*P* = 0.153), intermediate versus low (*P* = 0.666). (3) Total duration: high versus low (*P* = 0.415), intermediate versus low (*P* = 0.887). Data not shown.

### 3.2 Mode of delivery

The adjusted cross‐sectional multivariable associations between sleep variables and mode of delivery are presented in detail in the Supporting Information Appendix. In late pregnancy, habitual nocturnal awakenings were associated with higher odds of vacuum extraction delivery (aOR 1.45, 95% CI: 1.07–1.96, *P* = 0.018) and planned cesarean section (aOR 1.71, 95% CI: 1.01–2.89, *P* = 0.042). Short sleep duration (<6 h) was linked to an increased likelihood of unplanned cesarean section (aOR 2.57, 95% CI: 1.15–5.72, *P* = 0.021). Poor general sleep quality was associated with higher odds of both unplanned (aOR 1.68, 95% CI: 1.17–2.41, *P* = 0.005) and planned cesarean sections (aOR 2.60, 95% CI: 1.52–4.46, *P* < 0.001). At delivery, poor sleep quality remained associated with planned cesarean section (aOR 1.99, 95% CI: 1.04–3.82, *P* = 0.037).

In longitudinal analyses, persistent high or intermediate insomnia scores throughout pregnancy were not associated with the mode of delivery after adjustment (persistent high vs. low *P* = 0.670; intermediate vs. low *P* = 0.441). The same applied to sleepiness scores (persistent high vs. low *P* = 0.903; intermediate vs. low *P* = 0.576). Data not shown.

## 4 DISCUSSION

This study is the first to examine the association between maternal sleep across the entire pregnancy and delivery outcomes. We observed that deteriorating sleep was linked to the mode of delivery, even after adjustment for potential confounders. Poor general sleep quality, frequent nocturnal awakenings, and shorter sleep duration, particularly in late pregnancy, were associated with a higher likelihood of operative delivery, including cesarean sections and vacuum‐assisted vaginal births. However, persistent insomnia and sleepiness symptoms throughout pregnancy were not related to operative deliveries. Contrary to our hypothesis, poor maternal sleep was not associated with the duration of vaginal delivery.

### 4.1 Sleep and the mode of delivery

Previous cross‐sectional studies have also reported associations between insomnia or short sleep and increased cesarean section risk, although findings have been variable across studies. Our results were consistent with those of an American study^15^ that evaluated sleep in 131 mothers during the third trimester. Using both objective (wrist actigraphy) and subjective sleep measurements, they found that short sleepers (<6 h), one month before delivery, were 4.5 times more likely to deliver with cesarean section. In that study, however, maternal mental health symptoms were not accounted for, and sleep was assessed exclusively among healthy primiparous mothers. Two Iranian studies including 88 and 457 mothers, respectively,^14^, ^18^ also found that short sleepers during late pregnancy were more likely to have cesarean sections, but again, no information concerning mental symptoms, fear of vaginal delivery, or wish for cesarean section was collected. In a Malaysian study with 216 mothers, short sleep duration was associated with cesarean section in mothers with induction of labor.^16^ Similarly, in a Chinese study with 688 healthy mothers, poor sleep quality was associated with an increased likelihood of cesarean section.^17^

On the contrary, however, an American study with 88 mothers^25^ and a Canadian study with 624 women^21^ could not show any associations between sleep disturbances and risk for cesarean section. In those studies, mental problems were taken into account, and in the latter study,^21^ also a wish for cesarean section. In our earlier study in another birth cohort, sleeping disturbances were associated only with a higher probability of planned cesarean section, but not with unplanned cesarean section.^26^ In that study, we used qualified questionnaires and controlled the data with several confounding factors, including anxiety and depressiveness, similarly to the present study.

Because sleep disturbances are related to a lower tolerance for pain,^27^, ^28^ it can be hypothesized that mothers experiencing poor sleep have diminished resilience in coping with the stress, pain, and physical demands of childbirth, thereby increasing their risk of operative delivery. Sleep disturbances and mental symptoms like anxiety and depressiveness are strongly interrelated during pregnancy,^29^ which in turn may increase the risk and need or desire for cesarean section.^26^ In addition, somatic complications arising from impaired sleep may also contribute to these outcomes.

Still, the medical decision of performing a cesarean or other operative delivery is always multifactorial, circumstantial, and individual. Therefore, it is difficult to determine the precise impact of poor sleep on the clinical risk of cesarean section. Moreover, unexpected intrapartum events, such as acute fetal or maternal distress, often exert a substantial influence on the mode of delivery. These factors are inherently challenging to anticipate and were consequently not accounted for in this study.

### 4.2 Sleep and the duration of delivery

As for the relationship between maternal sleep and duration of vaginal delivery, earlier studies mentioned above^14^, ^15^, ^18^ have reported that poor maternal sleep quality and short sleep are associated with a longer duration of delivery. For instance, in the Iranian study,^18^ mothers who slept less had longer deliveries, with a mean duration of 11.8 h in the poor sleep group compared to 8.8 h in the good sleep group. However, in that study, oxytocin was commonly administered, the cesarean section rate was high, and the results were not adjusted for parity or oxytocin use. In our analysis, we did not observe any consistent or replicable associations. Although in mid‐pregnancy poorer general sleep quality was linked to a slightly longer first stage and total duration of labor, the clinical relevance of this finding is uncertain. Moreover, we were unable to replicate this association at other time points during pregnancy. Given the large number of analyses performed, this result is likely to represent a chance finding.

### 4.3 Sleep across the pregnancy and delivery

Sleep disturbances, particularly varying insomnia symptoms, are experienced by the majority of pregnant women throughout pregnancy due to multiple contributing factors.^2^, ^11^ However, contrary to our hypothesis, we did not identify convincing evidence of an association between persistent insomnia or persistent sleepiness and delivery outcomes, as the significant findings emerged only in the cross‐sectional analyses of third trimester data.

It can be hypothesized that although sleep disturbances are highly prevalent in pregnancy, compensatory, adaptive, or counteractive mechanisms may buffer the impact of chronic sleep disruption. Sleep is regulated by multiple hormones, including cortisol, prolactin, melatonin, and growth hormone, all of which rise substantially during pregnancy.^30^, ^31^ A high level of progesterone also acts as a respiratory stimulant, helping to ensure that nocturnal ventilation remains adequate.^32^ These hormones help to ensure that sleep, although fragmented, remains deep and restorative, thereby preventing the accumulation of significant sleep deprivation.^31^, ^33^, ^34^, ^35^, ^36^ Also, behavioral changes occur, and mothers can extend bedtimes or take naps more often.

Chronic sleep deterioration may activate the sympathetic nervous system or the hypothalamic–pituitary–adrenal (HPA) axis, thereby increasing the risk of pregnancy complications or other health issues that could ultimately necessitate cesarean delivery.^8^, ^10^ Furthermore, systemic low‐grade inflammation has been proposed as a potential causal mechanism linking sleep disturbances to pregnancy complications,^10^, ^37^ which in turn may elevate the risk of instrumental delivery.^37^, ^38^

Most women in our study had uncomplicated pregnancies. Therefore, our findings may not clarify whether these adaptive or compensatory mechanisms are sufficient among mothers with pre‐existing conditions or pregnancy‐related complications.

### 4.4 Benefits and limitations of the study

To our knowledge, this is the first and largest study to examine the associations between maternal sleep and delivery outcomes across the entire pregnancy. As no specific maternal health exclusions were applied, the findings are likely generalizable to a typical pregnant population. Furthermore, we thoroughly adjusted our results with confounding factors, including mental symptoms and gestational weeks. Sleep data were collected prospectively using repeated questionnaires, capturing participants' subjective perceptions of sleep across pregnancy. Notably, prior studies have applied highly variable methods for assessing sleep disturbances, and structured sleep questionnaires such as those used in the present study have rarely been utilized.

However, no measurement of sleep architecture was carried out. The mismatch between subjective sleep quality and sleep architecture is known,^39^ yet conducting a study of this scale using polysomnography would have been extremely time‐consuming and not feasible in terms of cost‐effectiveness. Furthermore, at each assessment point, mothers retrospectively evaluated their sleep over the preceding month, which may have resulted in either overestimation or underestimation of sleep disturbances. Nonetheless, this potential bias was likely consistent across all participant mothers. Some mothers may have used pharmacologic or non‐pharmacologic treatments to alleviate sleep disturbances during pregnancy. Unfortunately, we did not have access to this information and therefore could not evaluate the potential association between such treatments and mode of delivery.

Concerning the mode of delivery, the cesarean section rate in our cohort was lower than what is normal in the Finnish population. This is explained by the exclusion of breech presentation and twin pregnancies. The overall cesarean rate in Finland was 16.0%–16.3% around the time of the survey 2010–2015.^40^ Furthermore, comparisons across studies are challenging, as the prevalence of instrumental deliveries—and particularly cesarean section rates—varies substantially between studies and countries, ranging from approximately 10% to as high as 55%.^17^, ^18^, ^21^ Nevertheless, the ultimate mode and, in some cases, the timing of delivery may largely reflect the clinical decisions and practices of healthcare professionals. Also, the duration of delivery is influenced by multiple factors, mechanisms, and circumstances, it is challenging to assess accurately. In addition, variation in the estimation and documentation of delivery duration may contribute to measurement inconsistency. We also observed that adjusting for confounding factors substantially attenuated the associations, suggesting that these factors play an important role in the course of delivery.

Although causality cannot be established in observational studies such as ours, the findings indicate that poor sleep should be more systematically recognized as a potential risk factor for delivery outcomes.

### 4.5 Comments

Effective management of pregnancy‐related sleep disturbances may have the potential to reduce risks associated with pregnancy and childbirth, although direct evidence linking treatment of maternal sleep issues to obstetric outcomes, such as mode of delivery, is currently lacking. Despite their high prevalence, sleep disturbances are often underestimated during antenatal care and regarded as transient conditions expected to resolve postpartum, which may result in missed opportunities for intervention. Routine screening, particularly in late pregnancy, could improve maternal care; however, treatment remains complex due to safety concerns limiting pharmacologic options.^41^, ^42^ Consequently, non‐pharmacologic approaches—such as cognitive behavioral therapy for insomnia and structured sleep hygiene interventions—have shown promise in alleviating pregnancy‐related sleep problems.^43^, ^44^ Future research should clarify the impact of systematic identification and management of sleep disturbances on maternal and perinatal outcomes to inform their integration into standard antenatal practice.

## 5 CONCLUSIONS

As maternal sleep deterioration is highly prevalent during pregnancy and has been associated with an increased likelihood of operative deliveries, routine individualized assessment of sleep quality should be considered during prenatal care. However, given that delivery outcomes are often influenced by unpredictable factors, and that the observed associations were attenuated after adjustment for confounding variables, these findings should not be interpreted as indicating substantial clinical risk. Nonetheless, they warrant attention, particularly in pregnancies complicated by maternal or fetal conditions. Early identification of sleep disturbances and provision of appropriate information, support, and, when indicated, treatment may contribute to more favorable delivery outcomes.

## AUTHOR CONTRIBUTIONS

Hilla Parttimaa: Principal investigator and wrote the manuscript. Hasse Karlsson and Linnea Karlsson: Leaders of the FinnBrain study, co‐investigators, and co‐writers. Laura Perasto: Statistician of the study. E. Juulia Paavonen and Päivi Polo‐Kantola: Co‐leaders of the present substudy, co‐investigators, and co‐writers.

## FUNDING INFORMATION

This study was financially supported by the Finnish State Grants for Clinical Research (VTR) (P.P‐K.), Research Council of Finland #308588 and #342747 (E.J.P.), Research Council of Finland #308589, #342748, Signe and Ane Gyllenberg Foundation, Finnish State Grants for Clinical Research (VTR) (L.K) and the Research Council of Finland #325292, Signe and Ane Gyllenberg Foundation, Finnish State Grants for Clinical Research (VTR) (H.K).

## CONFLICT OF INTEREST STATEMENT

The authors declare no conflicts of interest.

## Supporting information


**Appendix S1.** Supporting Information.

## Data Availability

Research data are not shared.
