# Supplementary material for: Duration and mode of delivery: Does maternal sleep matter?
Source: Int J Gynaecol Obstet. 2025 Nov 20;173(2):829–38. doi: 10.1002/ijgo.70673 (PMC13094697; doi:10.1002/ijgo.70673)
Supplement: Supplementary file 1 — Appendix S1. Supporting Information. [file IJGO-173-829-s001.docx]

**Appendix Table 1a.**

**Appendix Table 1b.**

**Appendix Table 1c.**

**Appendix Table 1d.**

**Appendix Table 2a.**

**Appendix Table 2b.**

**Appendix Table 2c.**

**Appendix Table 2d.**
